# The zeaxanthin epoxidase is degraded along with the D1 protein during photoinhibition of photosystem II

**DOI:** 10.1002/pld3.185

**Published:** 2019-12-01

**Authors:** Stephanie Bethmann, Michael Melzer, Nadine Schwarz, Peter Jahns

**Affiliations:** ^1^ Plant Biochemistry Heinrich‐Heine‐University Düsseldorf Düsseldorf Germany; ^2^ Leibniz Institute of Plant Genetics and Crop Plant Research (IPK) Gatersleben Germany

**Keywords:** D1 turnover, photoinhibition, photosystem II, xanthophyll cycle, zeaxanthin, zeaxanthin epoxidase

## Abstract

The xanthophyll zeaxanthin is synthesized in chloroplasts upon high light exposure of plants and serves central photoprotective functions. The reconversion of zeaxanthin to violaxanthin is catalyzed by the zeaxanthin epoxidase (ZEP). ZEP shows highest activity after short and moderate high light periods, but becomes gradually down‐regulated in response to increasing high light stress along with down‐regulation of photosystem II (PSII) activity. ZEP activity and ZEP protein levels were studied in response to high light stress in four plant species: *Arabidopsis thaliana*, *Pisum sativum*, *Nicotiana benthamiana* and *Spinacia oleracea*. In all species, ZEP protein was degraded during photoinhibition of PSII in parallel with the D1 protein of PSII. In the presence of streptomycin, an inhibitor of chloroplast protein synthesis, photoinhibition of PSII and ZEP activity as well as degradation of D1 and ZEP protein was strongly increased, indicating a close correlation of ZEP regulation with PSII photoinhibition and repair. The concomitant high light‐induced inactivation/degradation of ZEP and D1 prevents the reconversion of zeaxanthin during photoinhibition and repair of PSII. This regulation of ZEP activity supports a coordinated degradation of D1 and ZEP during photoinhibition/repair of PSII and an essential photoprotective function of zeaxanthin during the PSII repair cycle.

## 1 INTRODUCTION

The xanthophyll zeaxanthin (Zx) serves central photoprotective functions in land plants (Jahns & Holzwarth, 2012). It is involved in non‐photochemical quenching (NPQ) of excess light energy in photosystem II (PSII) and additionally acts as an antioxidant in the thylakoid membrane (Havaux, Dall'Osto, & Bassi, 2007; Havaux & Niyogi, 1999). Zx is formed in the de‐epoxidation reactions of the xanthophyll cycle (Jahns, Latowski, & Strzalka, 2009; Yamamoto, Nakayama, & Chichester, 1962) from violaxanthin (Vx). This reaction takes place in the thylakoid membrane and is catalyzed by the Vx de‐epoxidase (VDE) which is localized in the thylakoid lumen. VDE activity is strictly regulated by the thylakoid lumen pH and the VDE is activated at pH values < 6.0 (Hager, 1969; Pfündel & Dilley, 1993) along with the pH‐dependent qE component of NPQ (Briantais, Vernotte, & Picaud, 1979; Takizawa, Cruz, Kanazawa, & Kramer, 2007). This pH‐regulation ensures that Zx formation and NPQ induction occur only at light intensities that saturate the photosynthetic electron transport. The reconversion of Zx to Vx is catalyzed by the Zx epoxidase (ZEP) which is localized in the chloroplast stroma (Schwarz et al., 2015) and has a pH optimum of about 7.5 (Siefermann & Yamamoto, 1975). The exact regulation of ZEP activity, however, remains elusive.

NPQ comprises a number of different mechanisms, which have been identified mainly on basis of their different relaxation kinetics, and which have been termed qE, qT, qZ, and qI (Nilkens et al., 2010; Quick & Stitt, 1989; Walters & Horton, 1991). Zx is supposed to be directly or indirectly involved in most NPQ processes, including qE, qZ, and qI (Kress & Jahns, 2017; Nilkens et al., 2010). The pH‐regulated, so‐called energy‐dependent quenching qE is the most flexible NPQ component, related to its rapid regulation (within 1–2 min) in response to light‐dependent changes in the lumen pH. Under a wide range of natural conditions, qE represents the dominating NPQ component. Activation of qE is mediated by the PsbS protein, which acts as sensor of the lumen pH and induces conformational changes in the antenna of PSII through interaction with LHCII proteins (Correa‐Galvis, Poschmann, Melzer, Stühler, & Jahns, 2016; Sacharz, Giovagnetti, Ungerer, Mastroianni, & Ruban, 2017). Induction of the maximum qE capacity further requires the presence of Zx. Two general models for the role of Zx in qE have been developed during the past years: A direct role of Zx in qE is favored by Fleming and co‐workers (Ahn et al., 2008; Avenson et al., 2008; Holt et al., 2005), while an indirect allosteric role has been proposed by Horton, Ruban and co‐workers (Horton, Ruban, & Wentworth, 2000; Kana et al., 2016; Ruban & Horton, 1999). Since current models of qE quenching locate the quenching process to the internal binding sites L1 and L2, a direct function of Zx likely requires binding of Zx at these sites, whereas an indirect allosteric role might also be based on Zx binding at the periphery of antenna proteins. Recent work hypothesized binding of Zx to the periphery of antenna complexes (Xu, Tian, Kloz, & Croce, 2015) supporting rather an indirect role of Zx in qE. The qZ component of NPQ is activated/deactivated in the time range of 10–30 min and has been correlated with the dynamics of Zx synthesis/epoxidation (Dall'Osto, Caffarri, & Bassi, 2005; Nilkens et al., 2010). This component represents a sustained, pH‐independent (once Zx has been synthesized) form of quenching and is likely identical with the Zx‐dependent phase of photoinhibition described earlier (Jahns & Miehe, 1996; Leitsch, Schnettger, Critchley, & Krause, 1994; Thiele, Krause, & Winter, 1998). For the qZ component of NPQ, a direct function of Zx has been proposed for the monomeric antenna protein Lhcb5 (Dall'Osto et al., 2005). The photoinhibitory qI quenching is the slowest inducible and relaxing NPQ component, which becomes activated after prolonged illumination of plants at high light intensities. Photoinhibition involves the light‐induced damage of the PSII reaction center, which is accompanied by phosphorylation and proteolytic cleavage of the D1 protein (Aro, Virgin, & Andersson, 1993; Ohad, Kyle, & Arntzen, 1984). Recovery from photoinhibition requires degradation and re‐synthesis of D1. This repair cycle likely occurs in the stroma exposed region of the thylakoid membrane (Jarvi, Suorsa, & Aro, 2015) and involves degradation of the D1 protein by FtsH and Deg proteases (Kato, Miura, Ido, Ifuku, & Sakamoto, 2009; Kato, Sun, Zhang, & Sakamoto, 2012). The possible role of Zx in qI is unknown. Recent analyses of the dynamics of NPQ and xanthophyll conversion, however, brought evidence that Zx has no direct function in qZ and qI (Kress & Jahns, 2017).

Earlier work indicated that Zx epoxidation is kinetically correlated with recovery from photoinhibition (Jahns & Miehe, 1996; Verhoeven, Adams, & Demmig‐Adams, 1996) and stepwise down‐regulated with decreasing PSII activity under high light stress (Kress & Jahns, 2017; Reinhold, Niczyporuk, Beran, & Jahns, 2008). The molecular basis of this down‐regulation of ZEP activity is unclear. Recent studies provided evidence that ZEP activity is susceptible to redox regulation. Arabidopsis *ntrc* mutants, which are defective in NADPH thioredoxin reductase C (NTRC), accumulate higher levels of Zx than wild‐type plants upon illumination at non‐saturating light intensities (Naranjo et al., 2016). Although no redox modification of the ZEP was detectable in that work, reduction of sulfhydryl group of the ZEP protein by either NTRC or via thioredoxins (Trx) may be involved in the regulation of ZEP activity. The latter idea was strongly supported by recent analyses of Arabidopsis plants with silenced Trx m proteins (Da et al., 2017). Like *ntrc* mutants, also *trxm* mutants accumulate higher levels of Zx than wild‐type plants upon illumination at non‐saturating light intensities. Moreover, this work provided evidence for a redox modification of ZEP and a direct interaction of Trx m and ZEP (Da et al., 2017). These data underline, that the ZEP protein becomes (at least partially) inactive in darkness along with the oxidation of Trx, and requires light activation through the Trx system for full activity. The high light‐induced down‐regulation of ZEP activity might thus be based on redox regulation.

To investigate the comparative down‐regulation of PSII and ZEP activity and changes in the protein level of D1 and ZEP, we studied the inactivation of ZEP during photoinhibition in Arabidopsis, pea, tobacco, and spinach. Our data suggest a concomitant degradation of D1 and ZEP protein after severe light stress, indicating an important role of Zx in photoprotection of PSII during high light (HL)‐induced D1 turnover.

## 2 MATERIAL AND METHODS

### 2.1 Plant material and growth conditions

Arabidopsis (*Arabidopsis thaliana*, ecotype Col‐0), pea (*Pisum sativum,* cv. Kleine Rheinländerin), and spinach (*Spinacia oleracea*, cv. Polka) plants were grown in a greenhouse under short‐day conditions (8‐hr light/ 16‐hr darkness) at light intensities of about 100 µmol photons m^−2^ s^−1^. Tobacco (*Nicotiana benthamiana*) plants were grown in a greenhouse under long‐day conditions (16 hr light/8 hr darkness) at light intensities of about 150 µmol photons m^−2^ s^−1^. About 6–8 weeks old plants were used for all species, except for pea plants, which were harvested after 2–3 weeks. For all experiments, plants at the end of dark phase were used as a control.

### 2.2 Isolation of intact chloroplasts and thylakoid membranes

A total of 10–20 g leaf material was harvested from dark‐adapted plants, washed with water and stored for 1–2 hr at 4°C in the dark. Chloroplast isolation was carried out according to (Kley, Heil, Muck, Svatos, & Boland 2010). In short, leaf material was homogenized in isolation medium (1 mM MgCl_2_,1 mM MnCl_2_, 10 mM NaHCO_3_, 300 mM sorbitol, 20 mM HEPES/ KOH, pH 7.6, 5 mM EGTA, 5 mM EDTA) and filtered through 4 layers of gauze and 1 layer of nylon mesh (40 µm). After centrifugation (10 min, 2,000 *g*) through a 50% percoll cushion, the resulting pellet, which contained intact chloroplasts, was resuspended in isolation medium. For isolation of thylakoid membranes, intact chloroplasts were osmotically shocked by 1 min incubation in 5 mM MgCl_2_. After centrifugation for 5 min at 4,000 *g*, the pelleted thylakoid membranes were resuspended in a medium containing 5 mM MgCl_2_, 5 mM NaCl, 2 mM KH_2_PO_4_, 40 mM HEPES/NaOH pH 7.6, and 0.33 M sorbitol.

### 2.3 Infiltration with streptomycin

For infiltration with streptomycin (SM), leaf disks (1 cm diameter) were placed on a solution containing 3 mM SM and incubated for 1 hr in a desiccator. Control samples were infiltrated with H_2_O under the same conditions.

### 2.4 Determination of PSII activity

Leaf disks were placed on water and exposed to HL (1,000 or 2,000 µmol photons m^−2^ s^−1^) on a temperature‐controlled cuvette (20°C or 4°C) for up to 8 hr. After HL treatment, samples were transferred to LL (10–20 µmol photons m^−2^ s^−1^) for up to 16 hr. At indicated times, leaf disks were incubated for 5 min in darkness and the Fv/Fm ratio was determined with a Joliot‐type spectrophotometer (JTS‐10, BioLogic). Subsequently, leaf disks were frozen in liquid N_2_ and stored for pigment extraction at −20°C. In addition, three leaf disks were frozen and stored at −20°C for protein extraction.

### 2.5 Protein extraction and immunoblot analyses

Proteins of leaf samples were extracted with protein extraction buffer (1.6% SDS (w/v), 1 M urea, 50 mM Tris/HCl pH 7.6) by mortaring in an Eppendorf vial (1,000 rpm, Heidolph RZR 2051). For chloroplast and thylakoid samples, 10 µl were mixed with 30 µl protein extraction buffer. Subsequently, protein extracts were incubated at 70°C for 20 min and non‐soluble material was pelleted by 20 min centrifugation at 13 000 *g*. Protein concentrations were determined with the Bio‐Rad^®^ DC Protein Assay (Bio‐Rad) according to the manufacturers protocol.

For immunoblot analysis, proteins were separated by SDS‐PAGE according to (Laemmli, 1970) and subsequently transferred to a PVDF membrane (FluoroTrans^®^W PVDF 0.2 µm, Pall Life Sciences) in a semi‐dry blot chamber (Power Blotter, Thermo Scientific), applying the 3 buffer system as described (Kyhse‐Andersen, 1984). Incubation with the primary antibody was carried out either for 1 hr at room temperature (RT) or overnight at 4°C, incubation with the secondary anti‐mouse‐IgG antibody (Sigma‐Aldrich) for 1 hr at RT. The secondary antibody was visualized by chemiluminescence (PicoLucent™ Plus‐HRP; GBiosciences) and detected with the LAS‐4000 mini (GE Healthcare). The following polyclonal primary antibodies were used; anti‐AtZEP and anti‐NtZEP (commissioned work, Pineda Antibody), anti‐Lhcb2 (AS01003), anti‐D1 (AS05084) and anti‐RbcL (AS03037) (Agrisera Antibodies).

### 2.6 Pigment analyses

For pigment analyses, frozen leaf material was carefully mortared in an Eppendorf vial (100 rpm, Heidolph RZT 2051) upon addition of acetone. After short centrifugation (5 min, 17,000 *g*), extracts were filtered (0.2 µm pore size) and then used for reversed‐phase HPLC according to (Färber, Young, Ruban, Horton, & Jahns, 1997).

### 2.7 Light and transmission electron microscopy

Leaf disks were placed on water and exposed to HL (2,000 µmol photons m^−2^ s^−1^) on a temperature‐controlled cuvette (4°C) for 8 hr. For structural analysis, the central part of leaf disks (1 cm diameter) of dark acclimated state and 8 hr of HL exposure were cut into 2 mm^2^ pieces. For each plant species and each treatment, 3–4 leaf disks were used for combined conventional and microwave‐assisted fixation, substitution, resin embedding, sectioning, and microscopical analysis as described (Schumann, Paul, Melzer, Dörmann, & Jahns, 2017).

### 2.8 Statistical analysis

Statistical analyses of pigment composition and light utilization parameters were performed using R studio (version 1.1.463). ANOVA was used to test significant differences among genotypes. Variance homogeneity was tested by Levenes's test. The post hoc test (Tukey HSD) was performed for multiple comparison analysis. Statistical analyses of immunoblot staining intensities were done with Student's *t* test using Microsoft Office Excel 2010–2016 (Microsoft Corporation). Significant differences (*p* < .05) were marked by different letters.

## 3 RESULTS

### 3.1 Spinach and pea chloroplasts do not contain stroma‐localized ZEP protein

**Figure 1:**
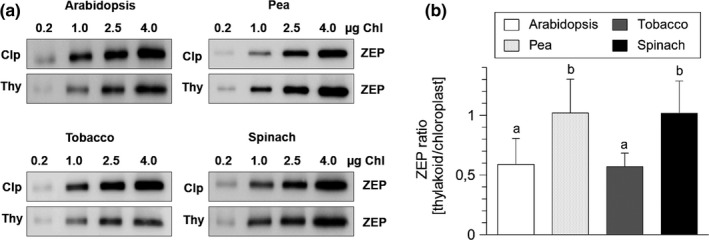
Abundance of ZEP protein in chloroplasts and thylakoid membranes. Intact chloroplasts and thylakoid membranes were isolated from dark‐adapted leaves. Proteins equivalent to the same Chl content of the corresponding chloroplast and thylakoid preparations were separated by SDS‐PAGE and ZEP abundance was assessed by immunoblotting. (a), Representative blots from 4 biological replicates. (b), Quantification of the ratio of ZEP content in thylakoids relative to chloroplasts. Mean values (± *SD*) of at least 2 technical replicates for each of 4 biological replicates (*n* = 8) are shown. Significant differences (Tukey HSD, *p* < .05) among the different species are indicated

Recent work has shown that ZEP protein in Arabidopsis chloroplasts is distributed between the thylakoid membrane (45%–50% of the total ZEP protein), the chloroplast stroma (45%–50%) and the envelope membrane (<10%) (Schwarz et al., 2015). The small fraction of ZEP protein associated with the envelope membrane is supposed to be involved in general carotenoid biosynthesis, while the thylakoid membrane‐bound fraction functions in the xanthophyll cycle (Schwarz et al., 2015). In contrast, the function of stroma‐localized ZEP protein is unknown. We investigated the existence of stroma‐localized ZEP protein in pea, spinach, and tobacco chloroplasts by comparing the protein content of intact chloroplasts with that of isolated thylakoid membranes. As shown in Figure 1, ZEP content of thylakoid membranes was clearly reduced to about 50% in comparison to ZEP content of chloroplasts in Arabidopsis and tobacco plants, supporting the existence of stroma‐localized ZEP protein in these two species. However, similar amounts of ZEP were found for thylakoid membranes and intact chloroplasts in pea and spinach (Figure 1), indicating that no stroma‐localized fraction of ZEP exists in these species. Obviously, the distribution of ZEP protein between the thylakoid membrane and the chloroplast stroma is not obligatory in chloroplasts of land plants. Moreover, the intensity of the bands detected for ZEP in chloroplasts was similar among the four species. Provided that the affinity of the antibodies to the ZEP protein is similar in each species, this indicates similar total levels of ZEP in chloroplasts of each species.

### 3.2 ZEP activity is inhibited by high light intensities

**Figure 2:**
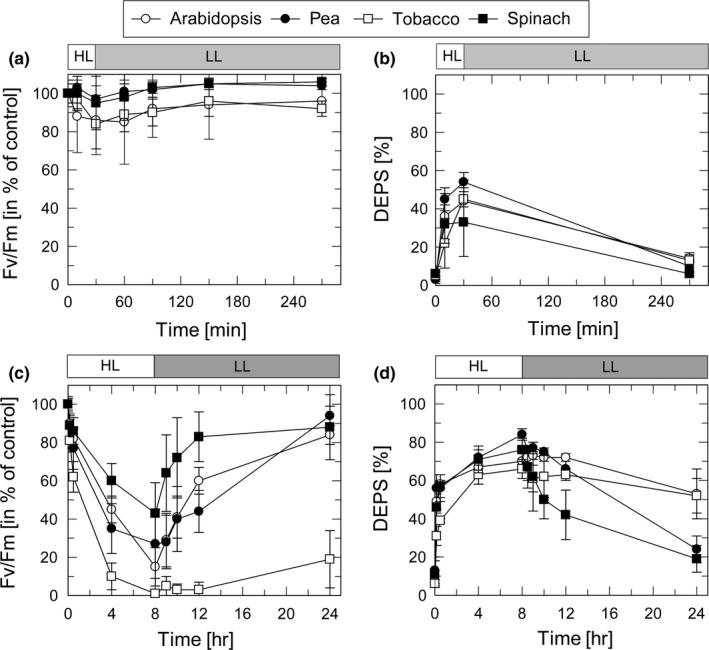
Dynamics of PSII and ZEP activity during and after HL exposure. Detached leaves from dark‐adapted plants were infiltrated with H_2_O and floated on water in a temperature‐controlled cuvette. Leaves were exposed to high light (HL) for 30 min at 1,000 µmol photons m^−2^ s^−1^ and 20°C (a,b) or for 8 hr at 2,000 µmol photons m^−2^ s^−1^ and 4°C (c,d). Subsequently, leaves were transferred to low light (LL, 10–20 µmol photons m^−2^ s^−1^) at 20°C for 4 hr (a,b) or 16 hr (c,d). PSII activity was derived from measurements of the Fv/Fm ratio (a,c) and ZEP activity from HPLC analysis of the de‐epoxidation state (DEPS) of the xanthophyll cycle pigments (b,d). DEPS [%] = (Zx + 0.5Ax)/(Vx + Ax +Zx) × 100. Data represent mean values ± *SD* from 8 (a,c) or 4 (b,d) independent measurements

**Figure 3:**
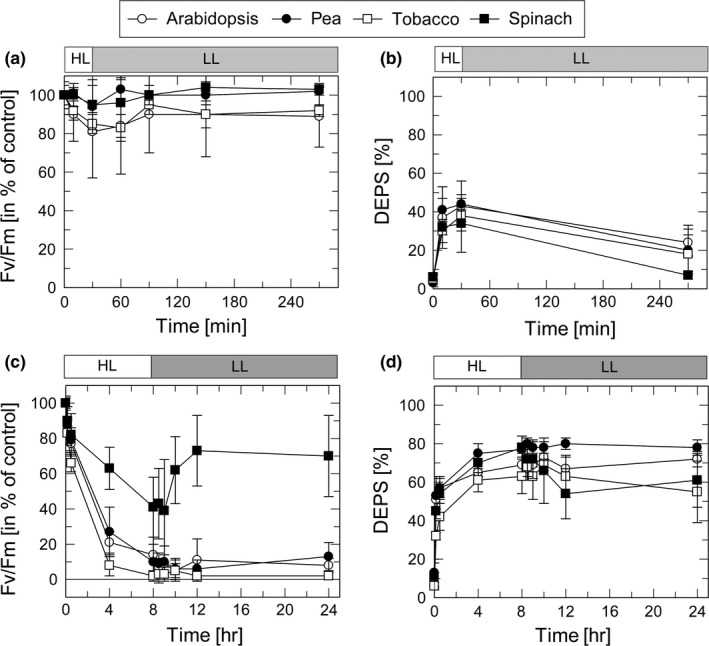
Impact of streptomycin HL‐induced inactivation of PSII and ZEP. Detached leaves from dark‐adapted plants were infiltrated with 3 mM SM and floated on water in a temperature‐controlled cuvette. Leaves were exposed to high light (HL) for 30 min at 1,000 µmol photons m^−2^ s^−1^ and 20°C (a,b) or for 8 hr at 2,000 µmol photons m^−2^ s^−1^ and 4°C (c,d). Subsequently, leaves were transferred to low light (LL, 10–20 µmol photons m^−2^ s^−1^) at 20°C for 4 hr (a,b) or 16 hr (c,d). PSII activity was derived from measurements of the Fv/Fm ratio (a,c) and ZEP activity from HPLC analysis of the de‐epoxidation state (DEPS) of the xanthophyll cycle pigments (b,d). DEPS [%] = (Zx + 0.5Ax)/(Vx + Ax +Zx) × 100. Data represent mean values ± *SD* from 8 (a,c) or 4 (b,d) independent measurements

The HL sensitivity of PSII and ZEP activity in the four species was studied on basis of changes in the maximum PSII efficiency as derived from the Fv/Fm ratio (for PSII activity) and the de‐epoxidation state (DEPS) of the xanthophyll cycle pigments (for ZEP activity). The response of plants to moderate HL stress (30 min at 1,000 µmol photons m^−2^ s^−1^ and 20°C) and severe HL stress (8 hr at 2,000 µmol photons m^−2^ s^−1^ and 4°C) was determined for leaves infiltrated either with H_2_O (Figure 2) or 3 mM streptomycin (SM) (Figure 3), an inhibitor of protein synthesis in chloroplasts.

Moderate HL stress (30 min at a light intensity of 1,000 µmol photons m^−2^ s^−1^ and at 20°C) induced a reduction of Fv/Fm to values in the range from 85% to 95% of the dark Fv/Fm ratio (Figure 2a). Pea and spinach plants showed the lowest reduction of the PSII quantum yield (to about 95%) and a complete recovery during subsequent LL (20–30 µmol photons m^−2^ s^−1^) exposure for 4 hr. In contrast, Arabidopsis and tobacco plants exhibited a more pronounced reduction of Fv/Fm (to about 85%) and the recovery in LL was incomplete (Figure 2a). In parallel, DEPS of the xanthophyll cycle pigments Zx, antheraxanthin (Ax), and violaxanthin (Vx), calculated as (Zx + 0.5Ax)/(Vx + Ax+Zx) × 100, increased during the HL period to values of 35%–55% and decreased to values between 5% and 15% at the end of the LL phase (Figure 2b). While the increase of DEPS in HL is related to the conversion of Vx to Zx by VDE, the decrease of DEPS in LL reflects ZEP activity. Comparing the differences among the four plants species, pea plants showed highest and spinach plants lowest DEPS at the end of the HL phase, while DEPS at the end of the LL phase was similar in all species. This indicates similar ZEP activities in all species after short‐term HL treatment.

More severe HL stress (8 hr at a light intensity of 2,000 µmol photons m^−2^ s^−1^ and at 4°C) induced a strong reduction of Fv/Fm to values ranging from 15% in Arabidopsis to about 30% in pea and about 45% in spinach (Figure 2c). In tobacco plants, however, Fv was completely abolished, resulting in Fv/Fm values of nearly 0 at the end of the HL treatment. During 16 hr recovery at LL and 20°C, the Fv/Fm values recovered almost completely in pea, spinach and Arabidopsis, while in tobacco plants an increase to only 30% was determined. Under the same conditions, DEPS increased during HL exposure to values between about 60% (tobacco) and 80% (pea and spinach) (Figure 2d). Compared to the moderate light treatment (Figure 2b) the decrease of DEPS in the subsequent LL period was retarded, resulting in DEPS values of 25%–30% (pea and spinach) and 45%–55% (Arabidopsis and tobacco) at the end of 16 hr LL exposure. Hence, severe HL stress resulted in down‐regulation of ZEP activity, which was more pronounced in Arabidopsis and tobacco as compared to pea and spinach. Overall, these data support the correlation of HL‐induced inactivation of PSII and ZEP in all species, with tobacco and Arabidopsis plants being more sensitive to HL than pea and spinach plants.

### 3.3 ZEP activity is inhibited in the presence of streptomycin

To evaluate the impact of the inhibition of the D1 turnover on HL‐induced changes in PSII and ZEP activity, leaves were incubated prior to HL exposure with SM. Under moderate HL stress (30 min at a light intensity of 1,000 µmol photons m^−2^ s^−1^ at 20°C), PSII activity was not affected by SM treatment as obvious from the similar dynamics of PSII activity in the presence (Figure 3a) and in the absence (Figure 2a) of SM. Interestingly, however, ZEP activity was slightly reduced in the presence of SM (20%–25% DEPS at the end of the LL phase, Figure3b, compared to 5%–15% in the absence of SM, Figure 2b). Only in spinach plants, no inhibitory effect of SM was detectable.

Under severe HL stress (8 hr at a light intensity of 2,000 µmol photons m^−2^ s^−1^ at 4°C), the PSII activity was nearly completely abolished in all plant species after HL treatment, except for spinach plants, which retained a residual activity of about 30% at the end of HL exposure (Figure 3c). While PSII activity in spinach recovered to values of about 60% during subsequent exposure for 16 hr to LL, no increase of the Fv/Fm ratio was detectable for the other species. This reflects the efficient suppression of recovery of photoinhibited PSII due to inhibition of D1 synthesis by SM. Hence, the differences among the species in the sensitivity of PSII activity toward HL were no longer visible in the presence of SM. The incomplete inactivation of PSII after HL treatment, as well as the partial recovery of PSII activity during subsequent LL exposure, in the presence of SM in spinach leaves is likely related to an incomplete uptake of SM in chloroplasts of this species.

Strikingly, inhibition of PS recovery was accompanied by complete inhibition of ZEP activity (Figure 3d). While synthesis of Zx—and thus the increase of DEPS—during the HL period was not affected by SM (cf. Figure 2d), no decrease of DEPS was detectable during the entire subsequent 16 LL exposure (Figure 3d). Only spinach plants showed a slight decrease of DEPS from about 80% to 60% during the first 4 hr of recovery, in line with the incomplete inactivation of PSII by SM in this species (Figure 3c 9***). These data indicate that ZEP activity is efficiently inhibited in the presence of SM. To test, whether SM is a direct inhibitor of ZEP, SM was added to thylakoids membranes, isolated from pre‐illuminated plants (30 min, 1,000 µmol photons m^−2^ s^−1^). In none of the species any significant effect of SM on ZEP activity was detectable (Figure S1), suggesting that the strong inhibitory effect on SM in HL treated leaves is due to an indirect function of SM, likely related to the suppression of the PSII repair cycle.

### 3.4 ZEP protein is degraded in parallel with the D1 protein

**Figure 4:**
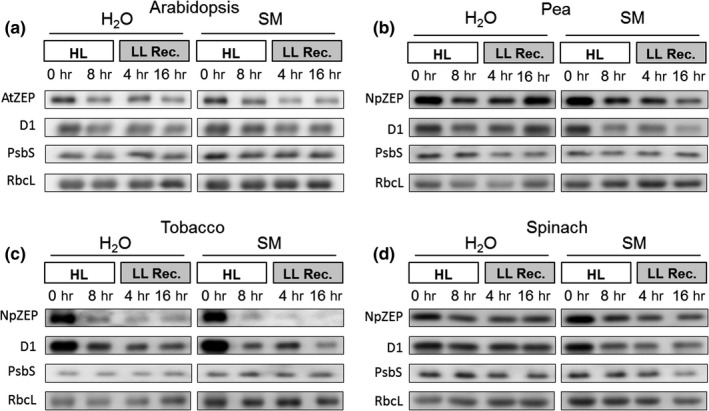
High light‐induced changes of the D1 and ZEP protein content. Detached leaves of Arabidopsis (a), pea (b), spinach (c) and tobacco (d) plants were vacuum‐infiltrated with H_2_O or 3 mM SM, as indicated in each panel. Leaves were exposed to HL for 8 hr at 2,000 µmol photons m^−2^ s^−1^ and 4°C and then transferred to LL (10–20 µmol photons m^−2^ s^−1^) for 16 hr. Total leaf protein extracts equivalent to 5 µg protein were separated by SDS‐PAGE. The abundance of ZEP and D1 protein, as well as of the large subunit of RubisCO (RbcL) was assessed by immunoblotting with specific antibodies. Representative blots from at least 3 biological replicates are shown

The impact of HL (2,000 µmol photons m^−2^ s^−1^ at 4°C) on the degradation of D1 and ZEP protein was determined by immunoblot analysis of leaf proteins extracted before and after 8 hr Hl treatment and during a subsequent recovery in LL (20 µmol photons m^−2^ s^−1^ at 20°C) (Figure 4). The PSII subunit PsbS and the large subunit of RubisCO (RbcL) were used as control proteins for thylakoid membrane proteins and stromal proteins, respectively. In absence of SM, moderate degradation of both D1 and ZEP protein was detectable in all plants (Figure 4, Figure S2). The most pronounced degradation of ZEP and D1 was found in tobacco plants, in accordance with the most pronounced HL sensitivity of PSII in this species (cf. Figures 2 and 3). The amount of PsbS or RbcL did not change under these conditions (Figure 4, Figure S2). In the presence of SM, D1 and ZEP degradation was enhanced in all species. Again, the most pronounced degradation was detected for tobacco plants (Figure 4d), while less pronounced degradation occurred in spinach plants (Figure 4b,c). The latter coincides with the highest resistance against HL determined for spinach (Figures 2 and 3). Again, the amount of PsbS and RbcL remained unchanged (Figure 4, Figure S2). The more pronounced degradation of ZEP together with D1 in the presence of SM suggests that ZEP degradation is directly coupled to D1 turnover. Hence, these data support the view that ZEP and D1 proteins degradation occurs in parallel in response to HL‐induced damage of PSII.

### 3.5 Species‐specific differences in HL sensitivity correlate with different VAZ pool sizes rather than with different light utilization capacities

Different HL sensitivities among the species could originate from different capacities of photosynthetic light utilization. Chl fluorescence analyses were applied to determine the light dependence of electron transfer and energy dissipation. The light dependence of the redox state of Q_A_ and P700 was derived from the parameters qL and Y_(ND)_, respectively, as determined with the DUAL PAM 101 fluorometer. For qL, Arabidopsis and pea plants showed a similar light response (50% reduction of Q_A_ at about 90 and 110 µmol photons m^−2^s^−1^, respectively), whereas spinach leaves exhibited half reduction of Q_A_ at significantly higher light intensities (about 170 µmol photons m^−2^ s^−1^ higher light intensities), and pea showed intermediate values (about 140 µmol photons m^−2^ s^−1^) (Figure S3a, Table 1).

**Table 1:** Light dependence of light utilization parameters derived from light response curves measured with the DUAL‐PAM 100 fluorimeter

Parameter		Arabidopsis	Pea	Tobacco	Spinach
qL	LI_50_	93 ± 23^a^	141 ± 18^bc^	109 ± 26^ab^	174 ± 16^c^
Y_(ND)_	LI_50_	100 ± 13^a^	117 ± 53^a^	127 ± 23^a^	197 ± 26^b^
NPQ	max	2.60 ± 0.32^a^	3.18 ± 0.44^b^	3.44 ± 0.26^bc^	3.83 ± 0.23^c^
	LI_50_	107 ± 12^a^	76 ± 55^a^	116 ± 31^a^	223 ± 45^b^
qE	max	2.08 ± 0.35^a^	2.73 ± 0.43^b^	2.98 ± 0.26^bc^	3.40 ± 0.24^c^

Note

Maximum values (max) or the light intensities at which half‐maximal values of the respective parameter were reached (LI_50_) are listed. Data represent mean values ± *SD* of 8 independent experiments. Superscript letters indicate significant differences (Tukey HSD, *p* < .05) among the different species.

Abbreviations: NPQ, total non‐photochemical quenching; qE, pH‐dependent component of NPQ; qL, fraction of oxidized Q_A_; Y_(ND)_, fraction of oxidized P700.

Similar trends were observable for the light response of the redox state of P700 (Figure S3b, Table 1). In this case, however, only spinach leaves required significantly higher light intensities for half‐maximal P700 oxidation (about 200 µmol photons m^−2^ s^−1^), whereas all other species showed similar values in the range from about 100–130 µmol photons m^−2^ s^−1^). Hence, the four species exhibit different light responses of the redox state of the electron transport chain, with spinach plants requiring the highest light intensities for reduction of Q_A_ and P700 oxidation. This became also evident from the light response of energy dissipation (Figure S3c, Table 1).

For NPQ induction, the light response was shifted to significantly higher light intensities in spinach leaves (half‐maximal NPQ at about 200 µmol photons m^−2^ s^−1^), as compared to the other species (about 80–120 µmol photons m^−2^ s^−1^). Moreover, spinach exhibited the highest maximum NPQ capacity among all species (Figure S3c, Table 1). These specific characteristics of spinach leaves might contribute to the lower HL sensitivity of this species compared to Arabidopsis and tobacco. However, the very similar light response determined for pea and tobacco plants (Figure S3, Table 1), which both showed pronounced differences in HL sensitivity, indicates that the light dependence of light utilization is not directly correlated with the overall HL sensitivity. Notably, also the maximum NPQ capacity, which was constituted predominantly by qE under these experimental conditions (Table 1), did not correlate with the susceptibility to photoinhibition. Therefore, photosynthetic light utilization is unlikely to be the critical determinant for different HL sensitivities of the four species.

We further analyzed the pigment content to obtain information about possible differences in the photosystem composition. The Chl a/b ratio, which can be used as an estimate for differences in the PSII antenna size, was lower in spinach plants as compared to all other species (Table 2). Although the difference was statistically not significant due to the high standard deviation, this indicates a slightly smaller PSII antenna size in spinach plants. Since pea plants showed a different HL response than Arabidopsis and tobacco, but a similar Chl a/b ratio, the PSII antenna size is likely not responsible for an altered sensitivity towards HL stress. However, the two species with lowest HL susceptibility (pea and spinach) exhibited a 2–3‐fold larger pool size of the xanthophyll cycle pigments Vx, Ax, and Zx (VAZ pool size, Table 2) and a higher convertibility of VAZ pigments during HL (DEPS, Figures 2 and 3, Table 2). These characteristics result in a much higher Zx content of pea and spinach plants compared to tobacco and Arabidopsis, which might explain the higher HL sensitivity of the latter two species. The VAZ pool size and DEPS might thus be critical determinants for the HL sensitivity of the species.

**Table 2:** Pigment composition

Parameter	Arabidopsis	Pea	Tobacco	Spinach
Nx	28 ± 3^ab^	31 ± 3^a^	25 ± 4^b^	32 ± 1^a^
Lut	92 ± 1^a^	119 ± 14^b^	89 ± 16^a^	91 ± 14^a^
VAZ	32 ± 3^a^	95 ± 19^b^	37 ± 13^a^	74 ± 7^b^
Chl a/b	2.92 ± 0.35^a^	3.10 ± 0.57^a^	3.09 ± 0.26^a^	2.66 ± 0.42^a^
DEPS_max_	70 ± 4^ab^	84 ± 3^c^	66 ± 5^a^	76 ± 6^bc^
Chl (a + b)/Clp	0.40 ± 0.10^a^	0.56 ± 0.11^a^	0.59 ± 0.08^a^	0.55 ± 0.02^a^
nmol Chl (a + b)/cm^2^ leaf area	28 ± 4^a^	38 ± 13^ab^	25 ± 4^a^	41 ± 11^b^

Note

The xanthophyll content (Lut, lutein; Nx, neoxanthin; VAZ, sum of xanthophyll cycle pigments) is given in relation to 1,000 Chl (a + b). The maximal de‐epoxidation state of the VAZ pigments (DEPS_max_) was derived from pigment analyses after 8 hr of illumination at 2,000 µmol photons m^−2^ s^−1^ (cf. Figure 2d). The data represent mean values ± *SD* with *n* > 20 for Nx, Lut, VAZ and Chl a/b; *n* = 8 for DEPS_max_ and *n* = 4 for Chl (a + b) per Chloroplast (Clp) and nmol Chl (a + b) per leaf area. Superscript letters indicate significant differences (Tukey HSD, *p* < .05) among the different species.

### 3.6 Leaf morphology and thylakoid membrane organization

**Figure 5:**
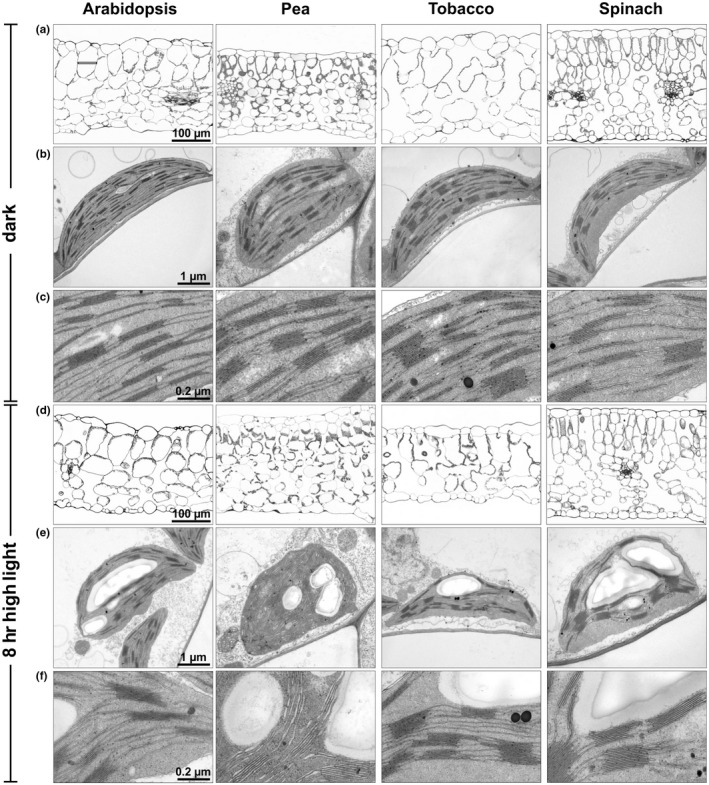
Leaf morphology and thylakoid membrane organization. (a,d) Light microscopic images of leaf cross‐sections. (b,e) Electron microscopic images of chloroplasts. (c,f) Thylakoid membrane organization. (a‐c) Dark acclimated leaves. (d‐f) Leaves after 8 hr of HL exposure. Representative images of at least 4 biological replicates are shown

Leaf morphology and thylakoid membrane organization was studied by light and electron microscopy to identify possible specific morphological characteristics that might be related to the more pronounced HL sensitivity of Arabidopsis and tobacco plants. Leaf thickness varied among the species between 200 and 300 µm, with pea plants showing the thinnest leaves and spinach the thickest ones (Figure 5a,d), and thus without specific structural properties of HL sensitive species. However, the two species with higher HL resistance, pea and spinach, were particularly characterized by smaller parenchyma cells (about 50 vs. 100 µm in Arabidopsis and tobacco) and more layers of parenchyma cells (Figure 5a,d). Thus, differences in leaf morphology might contribute to HL sensitivity. All species showed comparable chloroplast size and thylakoid membrane structures in the dark acclimated state, but pea chloroplasts were more round‐shaped compared to the oval‐shaped chloroplast of the other species (Figure 5b,e). However, whereas the amount of Chl per chloroplast was similar in all species, the two less HL sensitive species, pea and spinach, contained about 50% higher amount of Chl per leaf area (Table 2), which is in line with the increased number of parenchyma cells in these two species (Figure 5a,d). Please note, that this increase was only significant for spinach, due to the rather high standard deviation found in pea. Nevertheless, the Chl content per leaf area might thus also be a critical determinant for the HL sensitivity of the species. After 8 hr of HL exposure, leaf thickness was unchanged in all species except for tobacco plants, which exhibited a reduction of leaf thickness by about 30% (Figure 5d). This striking feature of tobacco leaves likely reflects the pronounced HL sensitivity of this species. Moreover, HL exposure induced structural changes of thylakoid membranes, particularly related to the stacking of membranes (Figure 5e,f). However, no general common response in relation to different HL sensitivity was detectable among the four species, but the two HL sensitive species (Arabidopsis and tobacco) tended to better preserve the grana structure of thylakoid membrane than the less HL sensitive ones (pea and spinach). Particularly pea chloroplasts showed a pronounced disordering of thylakoid membrane stacking, while grana stacks were rather increased in spinach chloroplast. Thus, HL‐induced reorganization of the thylakoid membrane might thus also contribute to the resistance against HL stress.

## 4 DISCUSSION

### 4.1 Regulation of ZEP activity

Zeaxanthin epoxidase activity is known to be down‐regulated upon increasing photoinhibition of PSII activity and hence photo‐oxidative stress in chloroplasts (Reinhold et al., 2008). Accordingly, ZEP activity was nearly completely inhibited in tobacco or strongly down‐regulated in the other species after 8 hr Hl treatment at 4°C, in parallel with PSII inactivation (Figures 2 and 3). Changes in ZEP protein amounts in response to HL stress have not been assessed so far. The HL‐induced degradation of ZEP shown in the present work thus indicates an irreversible HL‐induced damage of ZEP (analogous to D1). The HL‐induced down‐regulation of ZEP activity ensures that high levels of Zx are retained in response to prolonged HL stress to allow for efficient reactivation (or retention) of energy dissipation and thus photoprotection after intermediate LL phases. Hence, the inactivation of ZEP is understood as long‐term memory of photo‐oxidative stress (Jahns & Holzwarth, 2012). This regulatory principle also applies to in vivo conditions, since winter acclimation of evergreen plants was shown to be accompanied by retention of high Zx levels along with inactivation of PSII efficiency (Adams & Demmig‐Adams, 1995; Adams, Demmig‐Adams, Rosenstiel, Brightwell, & Ebbert, 2002; Adams, Demmig‐Adams, & Verhoeven, 1995; Öquist & Huner, 2003). These characteristics support an essential photoprotective function of Zx during photoinhibition of PSII. Notably, such a memory function of Zx also applies to short‐term down‐regulation of PSII efficiency in context with the pH‐regulated qE mechanism (Horton, Wentworth, & Ruban, 2005), which is also modulated by Zx. As the de‐activation of qE by the lumen pH is much faster than the reconversion of Zx to Vx, Zx is retained also in the short‐term, which ensures rapid reactivation of maximal qE capacity under rapidly fluctuating HL conditions.

The regulation of ZEP activity is thus central for photoprotection and operates at different time scales. However, the molecular basis of ZEP regulation is not fully understood. Recent work showed that ZEP activity is regulated in the short‐term by Trx (Da et al., 2017) and NTRC (Naranjo et al., 2016), implying an essential function of redox‐sensitive sulfhydryl groups of ZEP protein in regulation. On basis of the proposed redox regulation, it can be assumed that ZEP has low or no activity in the dark, and is fully activated under illumination through Trx mediated reduction of specific sulfhydryl groups. In contrast, the molecular basis of HL‐induced down‐regulation of ZEP activity is unclear. Related to the proposed redox regulation of ZEP activity, however, it is tempting to speculate that ZEP is inactivated by reactive oxygen species (ROS), which possibly irreversibly oxidize redox‐sensitive cysteine residues.

### 4.2 Degradation of ZEP protein

The observed HL‐induced degradation of ZEP protein indicates that it is irreversibly damaged upon high photo‐oxidative stress, possibly due to oxidation by ROS. Restoration of ZEP activity might thus require the degradation of inactive protein and import of newly synthesized protein. These characteristics resemble the general features of the well‐known HL‐induced D1 turnover. The very close correlation of the down‐regulation of PSII and ZEP activity and the parallel degradation of D1 and ZEP suggests a coordinated regulation of ZEP activity/degradation related to PSII inactivation/D1 degradation, which might involve an interaction of PSII and ZEP. ZEP is localized at the stroma side of the membrane and should have access to the stroma exposed regions of the membrane only, but not to the inner part of the grana region, where functional PSII is located. Since damaged PSII centers are supposed to migrate to the stroma exposed regions of the membrane, it is conceivable that ZEP can interact particularly with damaged PSII. However, on basis of non‐denaturing blue native gel electrophoresis, no co‐migration of ZEP and PSII was detectable in thylakoids from HL treated plants (Schwarz et al., 2015), suggesting that either no interaction or only a weak interaction of the two proteins exists. Nevertheless, an interaction of damaged PSII and ZEP might explain, why inactivation and degradation of ZEP protein is enhanced in the presence of SM. Since ZEP is encoded in the nucleus, ZEP synthesis should not be influenced directly by SM, suggesting that accelerated ZEP degradation in the presence of SM is rather related to the inhibited D1 turnover and thus PSII repair. Consequently, ZEP degradation might be triggered by the accumulation of non‐functional PSII, and hence under conditions when PSII repair cannot keep pace with PSII damage, either due to enhanced rates of PSII inhibition or due to reduced rates of PSII repair. The sensing of accumulation of non‐functional PSII by ZEP does not necessarily require an interaction of damaged PSII and ZEP but could also be related to a, so far unknown, factor involved in PSII repair or reassembly. Interestingly, ZEP activity was found to be affected by SM even under moderate HL stress, independent of an additional inhibitory effect on PSII activity. Since SM had no direct effect on ZEP activity, this supports the view, that the accumulation of a putative signaling factor can also be triggered independent of pronounced inactivation of PSII in the presence of SM. Consequently, inactivation (and likely also degradation) of ZEP seems to be not directly related to inactivation of PSII but rather to a functional PSII repair mechanism.

Irrespective of such a putative trigger factor, the down‐regulation/degradation of ZEP upon accumulation of damaged PSII implies an essential function of Zx for photoprotection during the PSII repair cycle. Such a function is supported by the characteristics of the Arabidopsis xanthophyll cycle mutants *npq1* and *npq2* (Niyogi, Grossman, & Björkman, 1998), which revealed an increased HL sensitivity of Zx deficient *npq1* (Havaux & Niyogi, 1999; Kalituho, Rech, & Jahns, 2007; Sarvikas, Hakala, Pätsikkä, Tyystjärvi, & Tyystjärvi, 2006) and a less pronounced HL sensitivity of Zx accumulating *npq2* (Dall'Osto et al., 2005; Kalituho et al., 2007) compared to wild‐type plants. Zx is known to be present in the thylakoid membrane either in association with antenna proteins or as non‐protein bound molecule in the lipid phase of the membrane. Since no binding of the Zx to the PSII reaction center has been reported so far, it is thus likely that non‐protein bound Zx serves as photoprotective xanthophyll during PSII repair. Such a function might be the basis for the shown qE‐independent function of Zx (Havaux & Niyogi, 1999). This view is further supported by well‐known increase of the VAZ pool size during long‐term HL acclimation of plants (Bailey, Horton, & Walters, 2004; Demmig‐Adams, Cohu, Muller, & Adams, 2012; Mishra et al., 2012; Schumann et al., 2017). Since long‐term acclimation to HL also involves the reduction of the PSII antenna size and thus of xanthophyll binding sites, it is very likely that a significant fraction of the additionally accumulated VAZ pigments is not bound to antenna proteins. Recent work challenged the view that formed Zx rebinds to the Vx binding sites of the PSII antenna proteins (Xu et al., 2015). Thus it can be speculated, that Zx is generally located at the surface of antenna proteins and by that may contribute to protection of the PSII reaction center.

### 4.3 Species‐specific differences in HL sensitivity

Plants of Arabidopsis and tobacco turned out to be more sensitive to HL than of pea and spinach. To determine putative specific physiological and morphological parameters of HL sensitivity, a comparative analysis of two more HL sensitive and two less HL sensitive species has been carried out. Based on our results, we suggest the following parameters to be crucial for the determination of HL sensitivity: (a) The presence of stroma‐localized ZEP, (b) Leaf morphology and thylakoid membrane dynamics, (c) The pigment composition (VAZ pool size and Chl content per leaf area).

The function of the stroma‐localized ZEP protein determined in Arabidopsis (Schwarz et al., 2015) (Figure 1) is unclear. Interestingly, a stroma‐localized fraction of ZEP was also found in tobacco chloroplasts, but not in the two less HL sensitive species, pea and spinach (Figure 1). Since the total amount of ZEP protein was found to be similar in all species (Figure 1), we conclude that the stroma‐localized fraction does not represent an additional pool of ZEP protein, but that binding of a fraction of ZEP to the thylakoid membrane is restricted. The ZEP protein binds to the stroma exposed region of thylakoid membrane and interacts with the membrane through hydrophobic interactions (Schaller, Wilhelm, Strzałka, & Goss, 2012; Schwarz et al., 2015). Species‐specific differences in the binding efficiency of ZEP protein to the thylakoid membrane might be due to differences in the properties of either the thylakoid membrane or the ZEP protein. Since the predicted amino acid sequences of the ZEP proteins in the different species show a high degree of identity, it seems unlikely that specific properties of the ZEP protein are responsible for the binding efficiency to the thylakoid membrane. It is unknown, however, which part of the protein is involved in binding to the membrane. It is further unclear, whether binding of ZEP requires a specific interaction with other proteins. In such a case, limited number of interactions partners present in the membrane might restrict ZEP binding to the membrane. Alternatively, different membrane properties could limit ZEP binding. For steric reasons, ZEP protein has only access to the stroma exposed regions of the membrane but not to the grana partitions. Thus, the relative portion of stroma exposed membranes might restrict ZEP binding as well. On basis of the EM analysis of thylakoid membrane organization (Figure 5), however, no obvious differences in thylakoid membrane organization were detectable among the different species under non‐stressed conditions. It is thus most likely, that other membrane characteristics are responsible for the reduced binding of ZEP protein and hence the occurrence of a stroma‐localized fraction of ZEP in Arabidopsis and tobacco.

At the level of leaf morphology, particularly the parenchyma cell size and the number of layers of parenchyma cells correlated with the HL sensitivity. The two species with higher HL resistance, pea and spinach, were particularly characterized by about 50% smaller parenchyma cells (about 50 µm in pea and spinach vs. 100 µm in Arabidopsis and tobacco) leading to more layers of parenchyma cells. The resulting light gradient within the leaf due to shading and light scattering might reduce light absorption in chloroplasts of inner layers of cells and hence induce reduced inactivation of PSII relative to the total number of chloroplasts. Earlier work further showed that a lower Chl content per leaf area is accompanied by higher susceptibility to photoinhibition (Patsikka, Kairavuo, Šeršen, Aro, & Tyystjärvi, 2002). Therefore, the lower Chl content per leaf area found here for Arabidopsis and tobacco plants (Table 2) could contribute to the higher HL sensitivity of these two species. Moreover, recent work showed that structural rearrangement of the thylakoid membrane is accompanied with light‐induced activation of qE (Schumann et al., 2017) and further regulates the balance between linear and cyclic electron transfer (Wood et al., 2018). Therefore, structural reorganization of membrane stacking is likely important for the activation of photoprotective mechanisms. The less pronounced thylakoid membrane reorganization in Arabidopsis and tobacco chloroplast in response to HL exposure compared to pea and spinach (Figure 5) might thus further determine the more pronounced HL sensitivity in these two species.

One of the most striking species‐specific feature of the two more HL resistant species, pea and spinach, is the strongly increased VAZ pool size (Table 2) compared to Arabidopsis and tobacco. The increased VAZ pool size has important consequences related to photoprotective properties. The large VAZ pool size provides a large fraction of non‐protein bound Zx in the lipid phase of the thylakoid membrane, which might contribute significantly to the protection of damaged PSII monomers during the PSII repair cycle in non‐appressed regions of the membrane, either due to ROS de‐activation in the lipid phase of the membrane or by promoting energy dissipation upon interaction with PSII reaction centers. ROS de‐activation might be of particular importance, because earlier work showed that D1 protein synthesis and hence repair of PSII is vulnerable to ROS (Kojima et al., 2007; Nishiyama et al., 2001), while contribution to energy dissipation would require an interaction of Zx with PSII, either at specific binding sites or at the surface of PSII reactions center or antenna proteins. Moreover, non‐protein bound Zx is supposed to affect the membrane properties with respect to fluidity and/or stability, similar to tocopherols (Havaux, 1998), and changes in the membrane fluidity are supposed to be involved in the PSII repair cycle (Goral et al., 2010; Yoshioka‐Nishimura, 2016). The less flexible thylakoid membrane structure in response to HL (Figure 5) observed in the two species with a small VAZ pool size, Arabidopsis and tobacco, thus strongly supports such a role of Zx. Since an increase of the VAZ pool size is a typical long‐term HL acclimation response of plants (Bailey et al., 2004; Demmig‐Adams et al., 2012; Mishra et al., 2012; Schumann et al., 2017), we propose that this parameter is an important factor determining the different HL sensitivities of the studied plant species.

## CONFLICT OF INTEREST

The authors declare no conflict of interest.

## AUTHOR CONTRIBUTIONS

P.J. conceived the project. P.J. and S.B. designed the experiments; S.B. performed most of the experiments; N.S. contributed to the experiments with Arabidopsis plants; M.M. performed the electron microscopy; P.J. wrote the article with contributions of all authors.
