# Supplementary material for: The zeaxanthin epoxidase is degraded along with the D1 protein during photoinhibition of photosystem II
Source: Plant Direct. 2019 Dec 1;3(11):e00185. doi: 10.1002/pld3.185 (PMC6885522; doi:10.1002/pld3.185)
Supplement: Supplementary file 1 [file PLD3-3-e00185-s001.pdf]

Figure S1

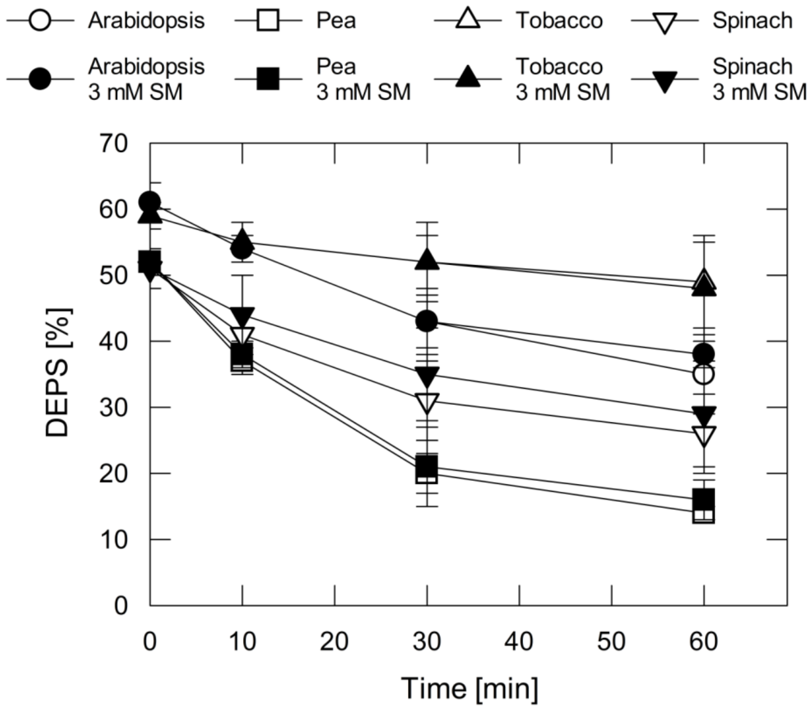

Figure S1. Impact of streptomycin on ZEP activity in isolated thylakoids. Thylakoid membranes were isolated from dark-adapted leaves pre-illuminated for 2 h at 1000 µmol photons m^{-2} s^{-1}. Thylakoid membranes equivalent to 100 µg Chl were suspended in epoxidation medium and gently stirred at room temperature and in the dark for up to 60 min. When indicated, 3 mM streptomycin (SM) was added. At indicated time, aliquots equivalent to 20 µg Chl were removed, and the de-epoxidation state (DEPS = (Zx+0.5Ax)/(Vx+Ax+Zx) x 100) of the xanthophyll cycle pigments was determined by HPLC analysis. Data represent mean values ± SD from 3 independent measurements.

Figure S2

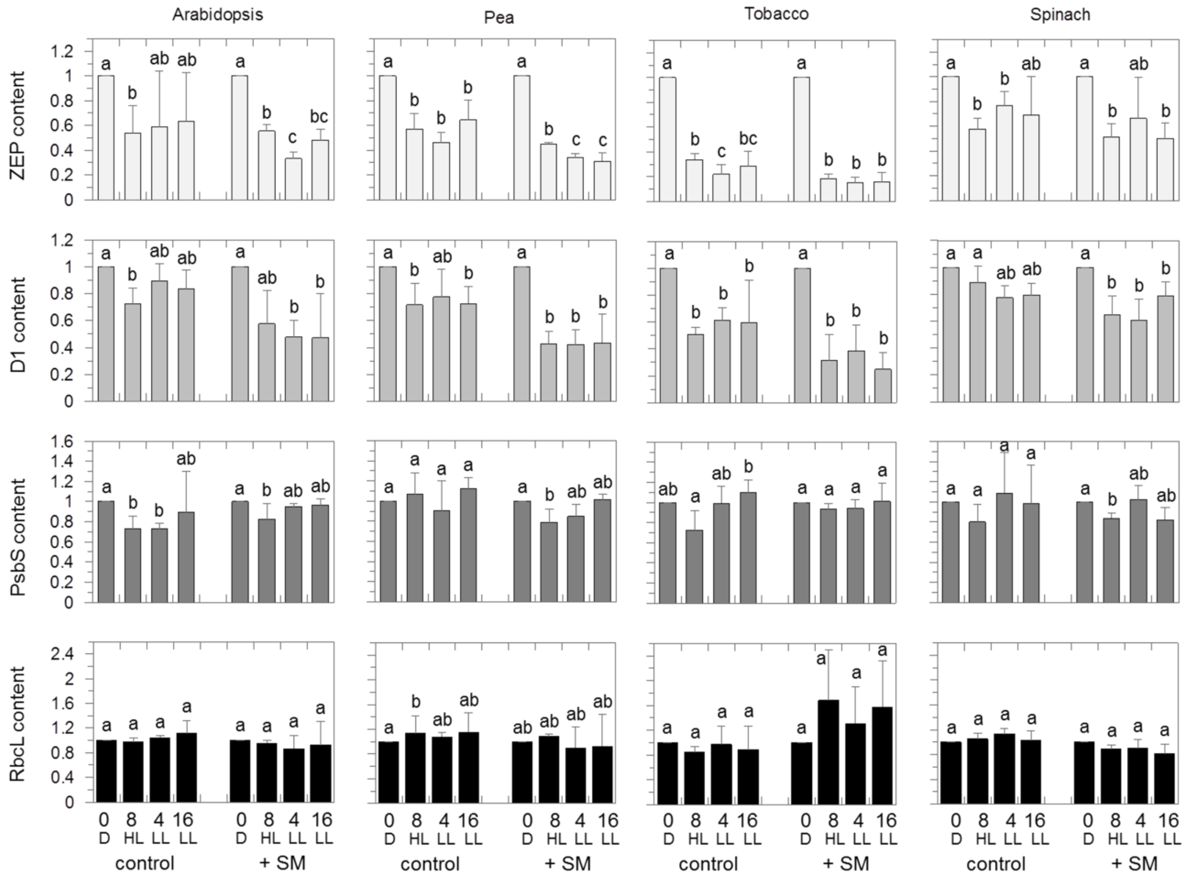

Figure S2. Quantification of HL-induced changes of the protein content. The relative abundance of ZEP, D1, RbcL and PsbS was estimated from immunoblot analyses with specific antibodies (representative blots are shown in Fig. 4). Signal quantification was carried out using the program ImageStudioLite. The signal intensities within each experiment (dark control (0, D); end of 8 hl HL (8, HL), and after additional LL recovery for 4 h (12, LL) and 12 h (24, LL)) are shown in relation to the control samples before onset of HL illumination (0, D), which were normalized to 1 in each case. Mean values (± SD) derived from 3 independent experiments (n = 3) are shown. Significant HL-induced differences in the abundance of the respective proteins (student's t-test, $p < 0.05$) are indicated for each species.

Figure S3

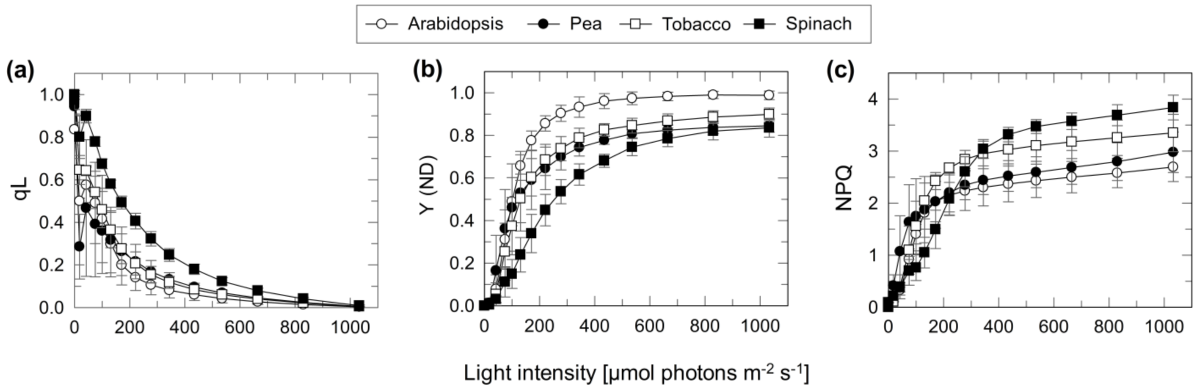

Figure S3. Light dependence of light utilization. The light response of the redox state of the plastoquinone pool (a), of the redox state of the PSI donor side (b) and of energy dissipation (c) was determined with the DUAL PAM 101 fluorometer. Before each measurement, plants were dark-acclimated for 2 h. Light response curves were measured from the lowest to the highest light intensity and leaves were acclimated to each light intensity for 2 min. Mean values ± SD of 4 independent measurements are shown.
