# Supplementary material for: The zeaxanthin epoxidase is degraded along with the D1 protein during photoinhibition of photosystem II
Source: Plant Direct. 2019 Dec 1;3(11):e00185. doi: 10.1002/pld3.185 (PMC6885522; doi:10.1002/pld3.185)
Supplement: Supplementary file 2 [file PLD3-3-e00185-s002.pdf]

We thank the reviewers for their valuable comments. Please find below our point by point response to the criticisms raised by reviewer 1:

REVIEWER COMMENTS:

Reviewer: 1

The authors have successfully answered my comments.

Response:
We thank for the positive evaluation of our revisions.

Reviewer: 2

General comments:

This manuscript reports the degradation of zeaxanthin epoxidase during photoinhibition of photosystem II (PSII). The authors found that the degradation of zeaxanthin epoxidase correlates with the degradation of the D1 protein and the sensitivity of PSII to photoinhibition in four plant species, Arabidopsis, pea, spinach, tobacco, and spinach. Based on the observations, they conclude that the degradation of zeaxanthin epoxidase, together with the degradation of the D1 protein, might play an important role in photoprotection and, in particular, in the repair of PSII. The manuscript is well written, and the discussion is very interesting. However, there are some concerns in this manuscript. My major concern is that it is not clear whether the manuscript aims at understanding of the general role of the degradation of zeaxanthin epoxidase or of species-specific differences in the sensitivity to high light. In the former case, the authors need to investigate the molecular mechanisms by which zeaxanthin epoxidase is degraded under photoinhibition conditions and also by which the degradation of zeaxanthin epoxidase is coupled with that of the D1 protein, perhaps using Arabidopsis. In this case, leaf morphology in Figure 5 might not be necessary. I am afraid that otherwise the purpose of this research would be vague. In the latter case, this manuscript appears to be a detailed description of different high-light sensitivities among four plant species.

Response:
We thank for the generally positive comments on the manuscript. We are aware that identification of the underlying molecular mechanism of zeaxanthin epoxidase (ZEP) degradation and its relation to D1 degradation is of high importance. However, this cannot be addressed by simple experiments but requires a thorough analysis of mutants (affected e.g. in PSII assembly and different proteases) and ZEP interaction partners. Such experiments are currently planned, but go beyond the scope of the present work.
We do not agree with the reviewer that the results on leaf morphology should be skipped. Actually, we included four species in this work to test whether ZEP regulation/degradation shows different responses in species with different high light sensitivity. Our results indeed proof, that the regulation/degradation of ZEP is strictly related to the high light-induced inactivation of PSII and/or degradation of D1. In this context, the investigation of factors, which might determine the high-light sensitivity is of general importance, with thylakoid membrane organization being one candidate among others.

Specific comments:

1. Abstract: Spelled out Zx. PSII/D1 does not make sense. Rephrase it, such as the D1 protein in PSII.

Response:
We rephrased the abstract accordingly.

2. Figure 1(b): The notes in the inset are incorrect. Exchange spinach and tobacco.

Response:
We thank for identification of this error and adapted Figure 1b accordingly.

3. Results, lines 120-122: Insert a reference into this sentence.

Response:
Done.

4. Results, lines 135-136: How can the authors compare the total levels of ZEP? The affinity of antibodies to ZEP might differ in different plant species.

Response:
We agree and added the aspect of antibody affinity.

5. Results, line 144: Spell out SM, as it appears first here.

Response:
Done.

6. Results, line 169: Is a pair of Arabidopsis and tobacco a typographical error of a pair of pea and spinach? Please check out.

Response:
The reviewer is right. We corrected this mistake.

7. Results, lines 186-187, Figure 3(c): Is PSII repaired in the presence of SM in spinach? I am wondering that SM might not have penetrated completely into chloroplasts of spinach leaves.

Response:
We agree with this interpretation and added this suggestion in the results section.

8. Discussion, the first paragraph: It seems that the downregulation of ZEP under photoinhibition has been reported. Are there any earlier works that report the degradation of ZEP under high light? If so, mention the earlier findings here. If not, emphasize the authors' findings.

Response:
Degradation of ZEP under high light has not been described so far, and we emphasized this aspect in the discussion.

9. Discussion, lines 356-362: These sentences seem to be contradictory to each other. The authors should make it clear whether ZEP activity is affected or unaffected by repair. I suppose that no signals might come out in the presence of SM, namely, in the absence of repair, if the activity or degradation of ZEP is coupled with repair.

Response:
We added a sentence to make clear that ZEP degradation is coupled with repair.

10. Discussion, page 18: As mentioned above, I feel that the results from leaf morphology makes this manuscript complicated and vague. I would suggest removing this part.

Response:
As pointed out in our response to the general comments, we do not agree that the data of leaf morphology complicate or weaken this manuscript, but rather represent one of different aspects that might determine high-light sensitivity.
